# Ectopic thyroid tissue in the breast mimicking a breast lesion: A rare case report

**DOI:** 10.1016/j.radcr.2026.07.107

**Published:** 2026-09-01

**Authors:** Hannah Holmesi, Mina Taghavi, Shaghayegh Rahmani, Penelope Moyle

**Affiliations:** a Department of Medicine, East Surrey Hospital, Redhill, RHI 5RH, United Kingdom; b Department of Radiology, Cambridge University Hospital, Cambridge, CB2 0QQ, United Kingdom; c Department of Emergency Medicine, MMS.C, Islamic Azad University, Mashhad, Iran

**Keywords:** Breast, Ectopic thyroid tissue, Mammography, Breast mass, SPECT-CT, Case report

## Abstract

Ectopic thyroid tissue is an uncommon developmental anomaly characterized by the presence of thyroid tissue outside its normal pre-tracheal site. This report describes a rare case of benign ectopic thyroid tissue in the breast of a 47-year-old woman after a history of Graves’ disease and benign left thyroidectomy. A core biopsy of the breast excluded malignancy from a breast cancer, thyroid and other metastatic diseases. Functional and anatomical imaging with I-123 SPECT CT and ultrasound of the neck were performed to identify all sites of thyroid tissue including ectopic sites. This case demonstrates the steps needed to understand the diagnosis and management of ectopic thyroid tissue in the breast and the need for a multidisciplinary approach with breast, nuclear medicine and endocrine teams for optimal management.

## Background

Ectopic thyroid tissue is defined as the presence of thyroid tissue outside its normal pre-tracheal location due to abnormal embryologic migration of the thyroid gland during early development [1].

The prevalence of ectopic thyroid tissue is estimated at approximately 1 in 100,000-300,000 in the general population, increasing to 1 in 4000-8000 among individuals with underlying thyroid disease [2]. The most common ectopic location is the lingual region (approximately 90% cases) while lateral cervical, mediastinal, pulmonary and abdominal locations are very rare [[2], [3], [4], [5]]. Occurrence within the breast is exceptionally uncommon, with only isolated case reports described in the literature [6].

Although ectopic thyroid tissue is generally benign and asymptomatic, malignant transformation is reported [7]. In rare cases thyroid malignancy, may metastasize to the breast [7,8].

This report presents a case of benign ectopic thyroid tissue mimicking a breast lesion. A breast core biopsy plus thyroid immunohistochemistry is needed to confirm benign ectopic thyroid tissue. Multidisciplinary working with nuclear medicine imaging and endocrine services are needed for the correct diagnosis and safe management of this rare condition.

## Case presentation

**Fig. 1:**
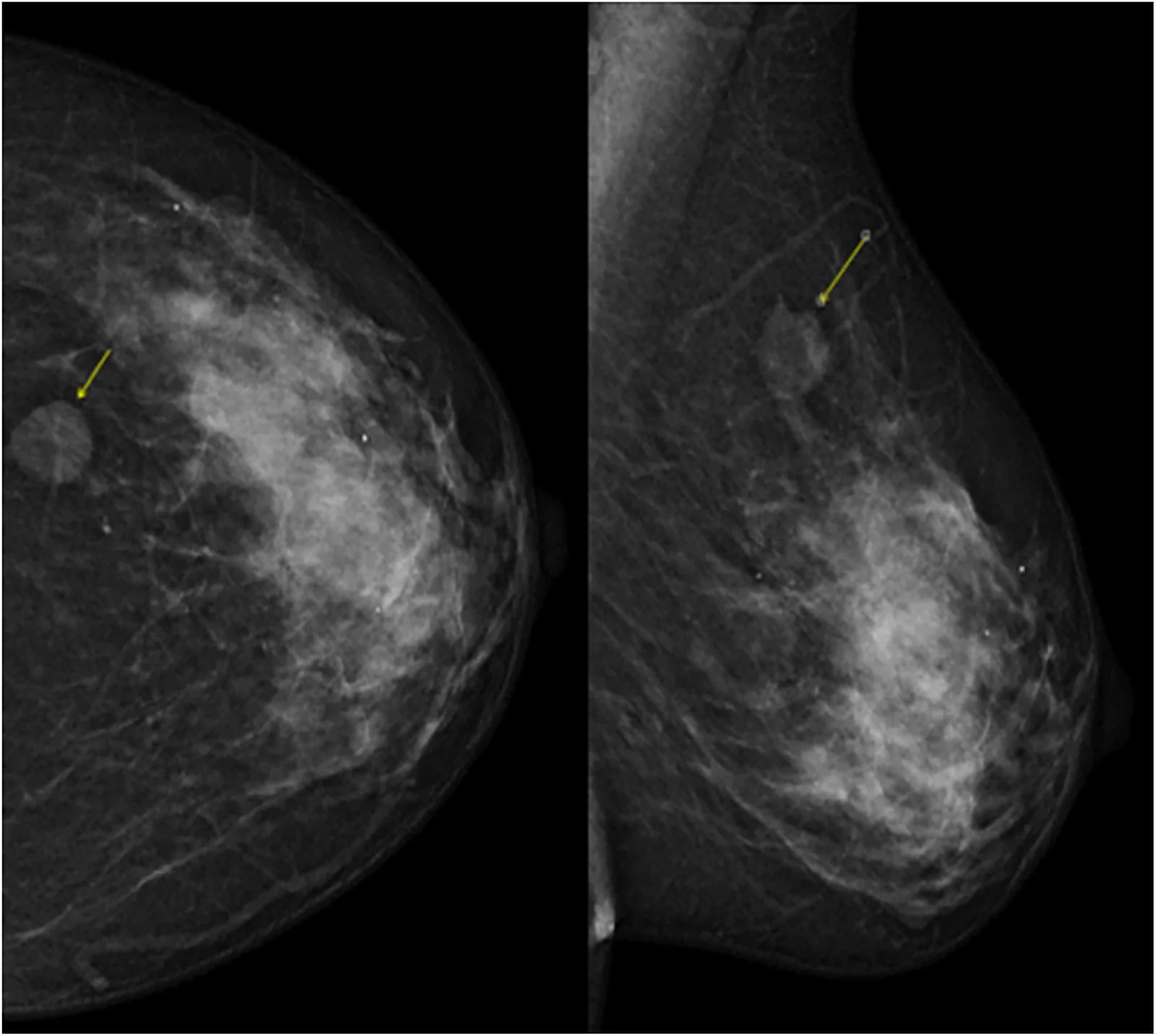
CC view (left) and MLO view (right) mammogram demonstrates a new 13 mm round circumscribed mass (arrow) in upper central left breast, BIRADS-3.

A 47-year-old woman was referred to the Breast Unit after her first routine screening mammograms identified a 13mm round, circumscribed mass in the left upper outer quadrant, (Fig. 1). This was classified as BIRADS 3 [9].

**Fig. 2:**
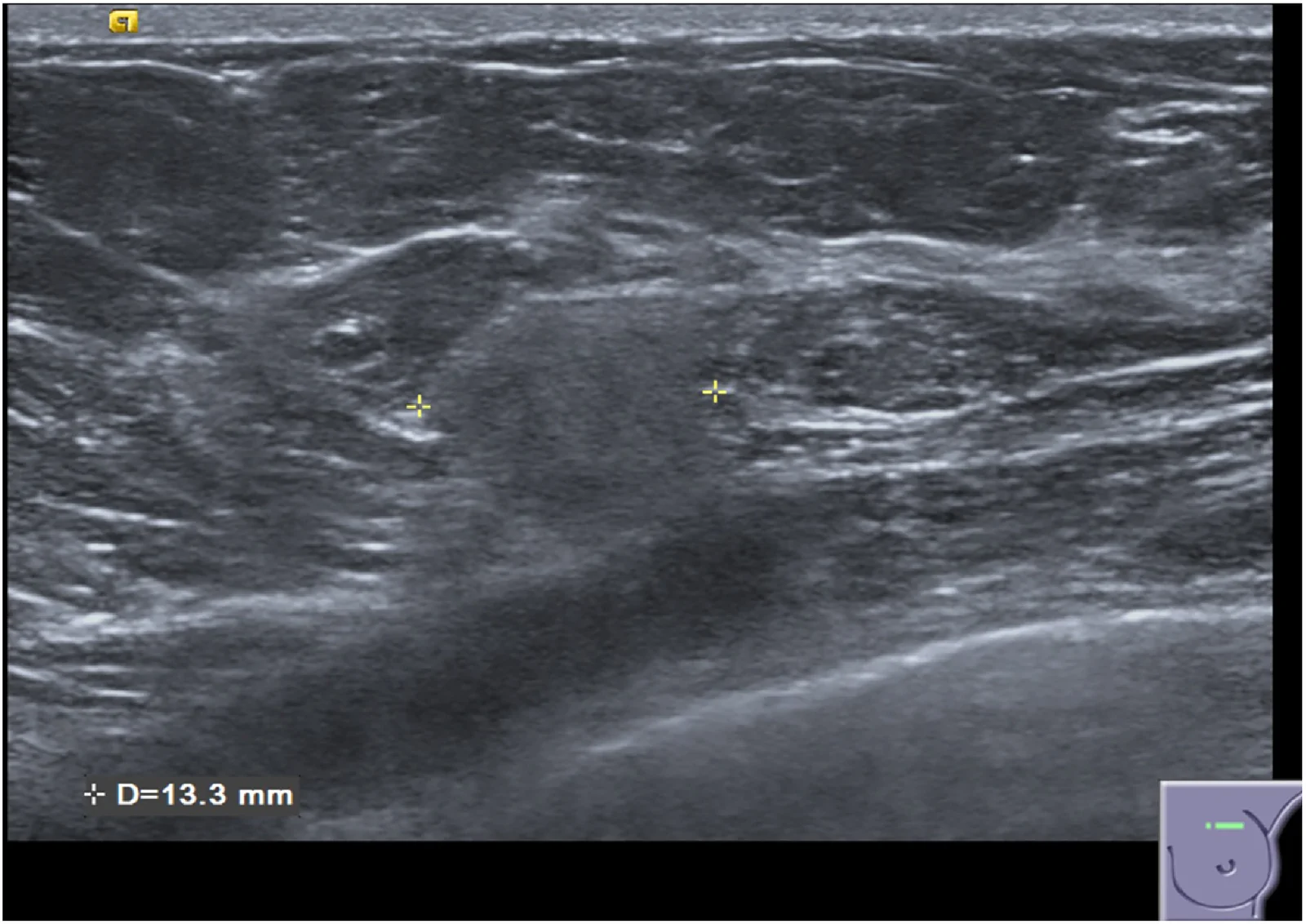
Ultrasound of the left breast demonstrates a hyperechoic 13 mm, round, partially circumscribed, partially indistinct isoechoic mass, BIRADS 4A.

Clinical examination was normal and the patient had not noticed an abnormality. Left breast tomosynthesis confirmed the original mammographic lesion. Ultrasound demonstrated a 13mm, round partially circumscribed, partially indistinct isoechoic mass, BIRADS 4A (Fig. 2). Normal left axillary lymph nodes were identified.

**Fig. 3:**
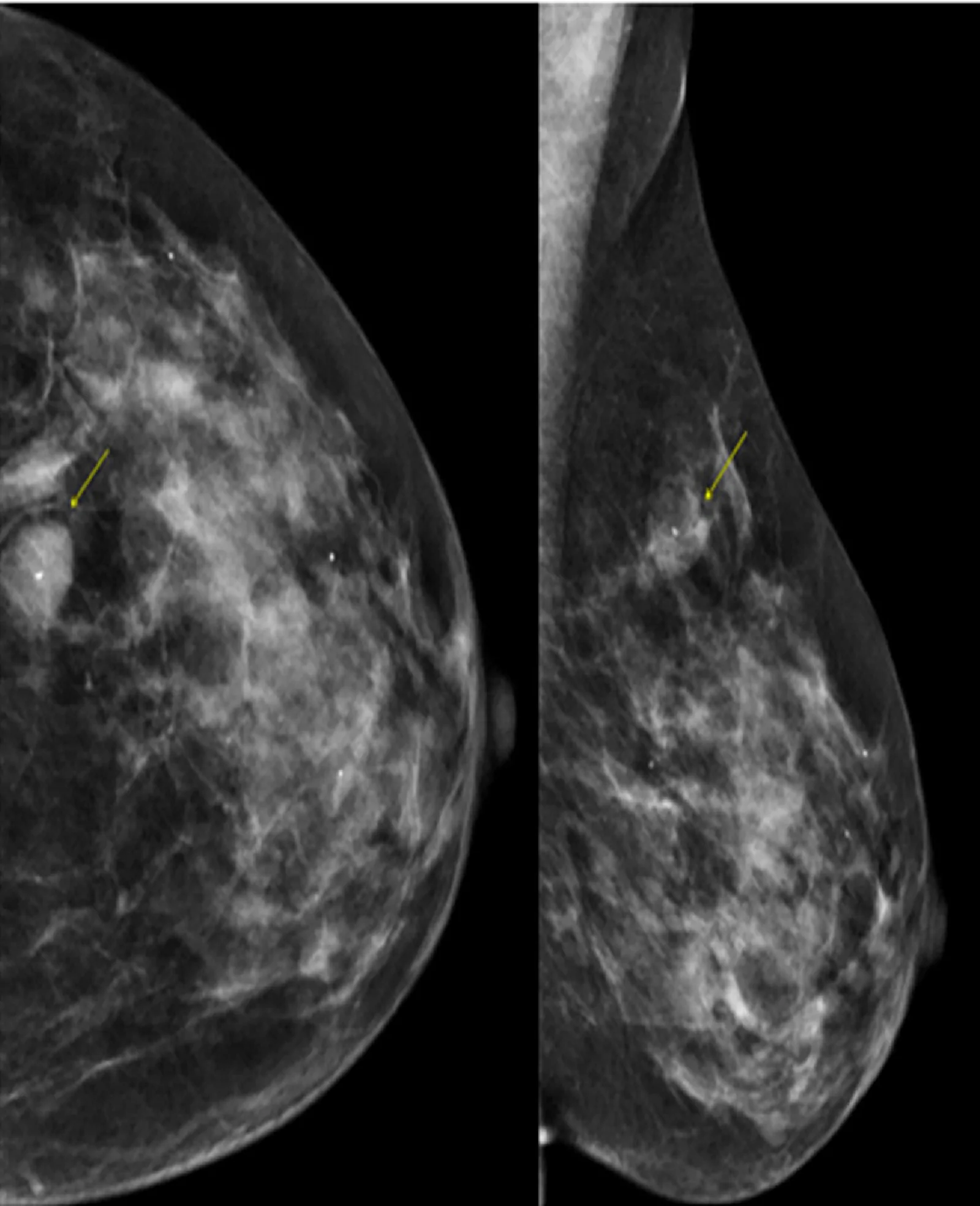
CC view (left) and MLO view (right) mammogram demonstrates the presence of a marker clip within the left breast lesion (arrow).

As the differential diagnosis was wide, and included an isoechoic malignancy, a biopsy was taken (2 × 14G cores). A radiological marker was placed at the biopsied site and its position confirmed concordant with the mammographic mass (Fig. 3).

Histopathology reported partially encapsulated benign thyroid tissue which was continuous with adjacent benign breast tissue. The thyroid tissue was composed of follicles containing colloid and lined by bland thyroid follicular epithelial cells which showed no nuclear features suggestive of papillary thyroid carcinoma. No necrosis, mitoses or vascular invasion was seen. Immunohistochemistry showed positive immunostains for TTF1, thyroglobulin and CD56, with negative HBME-1, Galectin 3 and BRAF. The CK19 showed patchy membranous and cytoplasmic positivity which can be seen in normal breast ductal and secretory cells. The MIB1proliferation index was low. This confirmed the thyroid tissue and surrounding sampled breast tissue was normal/benign, but excision of the lesion was recommended by the histopathologist for further characterization.

The outcome of the breast multidisciplinary team meeting was to refer to the endocrinologists for further advise on management before potential excision.

Subsequent history from the patient identified she had a history of Grave’s disease 30 years previously. She underwent subtotal thyroidectomy for benign disease and had been in remission with no current medication since. Current thyroid function was normal.

An ultrasound of the thyroid and fused radioiodine-123 SPECT/CT (Single-Photon Emission Computed Tomography (I-123 SPECT/CT) were performed to see if there was residual functioning thyroid tissue in the neck and if other areas of ectopic tissue were present.

**Fig. 4:**
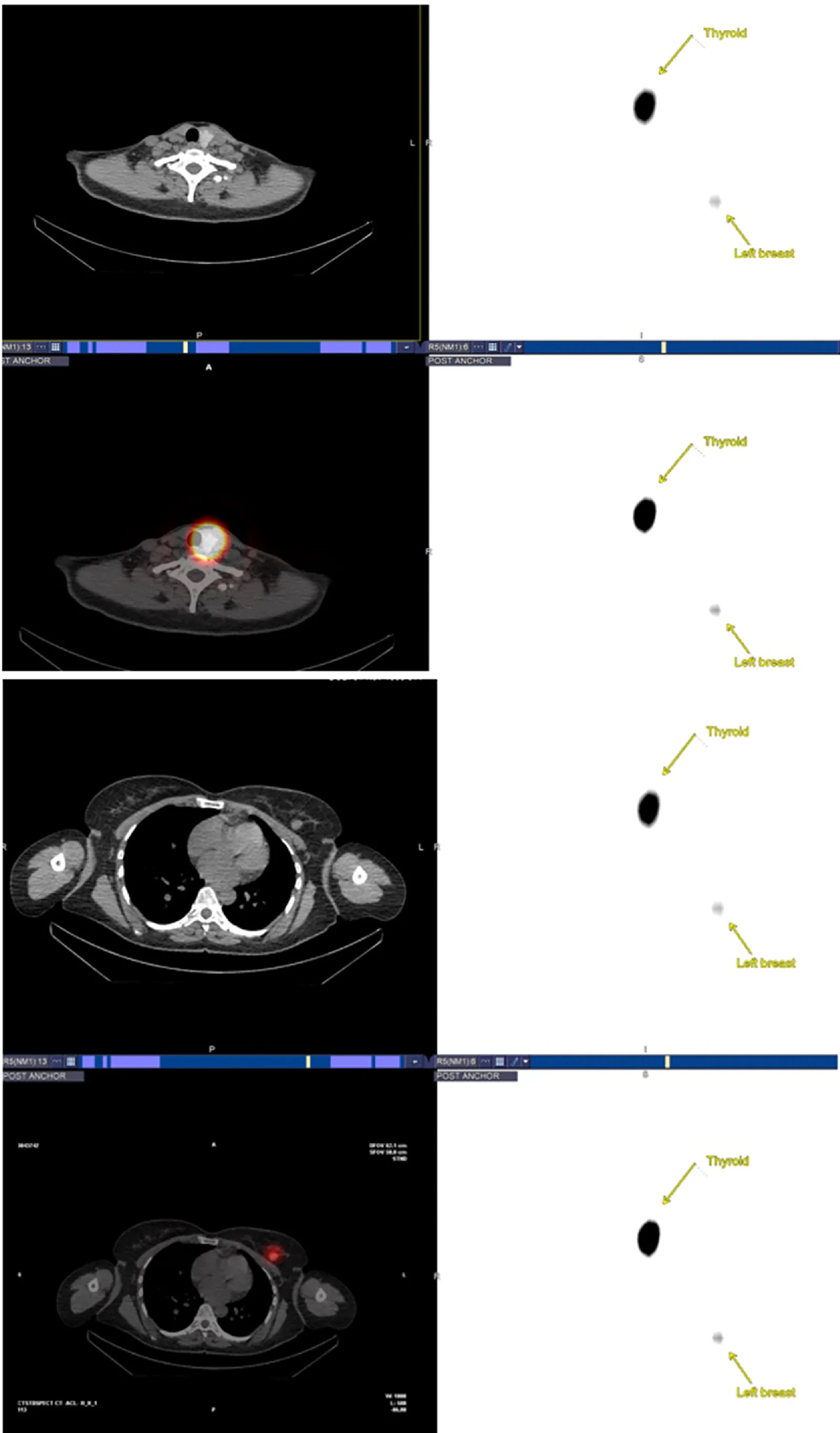
(A and B) I-123 SPECT CT identified accumulation of I-123 on the left lobe of the thyroid and within the ectopic tissue in left breast (A and B) confirming functioning thyroid tissue at both sites.

The I-123 SPECT CT identified accumulation of I-123 on the left side of the neck and within the left breast (Fig. 4A and B) showing functioning tissue only in the neck and breast.

**Fig. 5:**
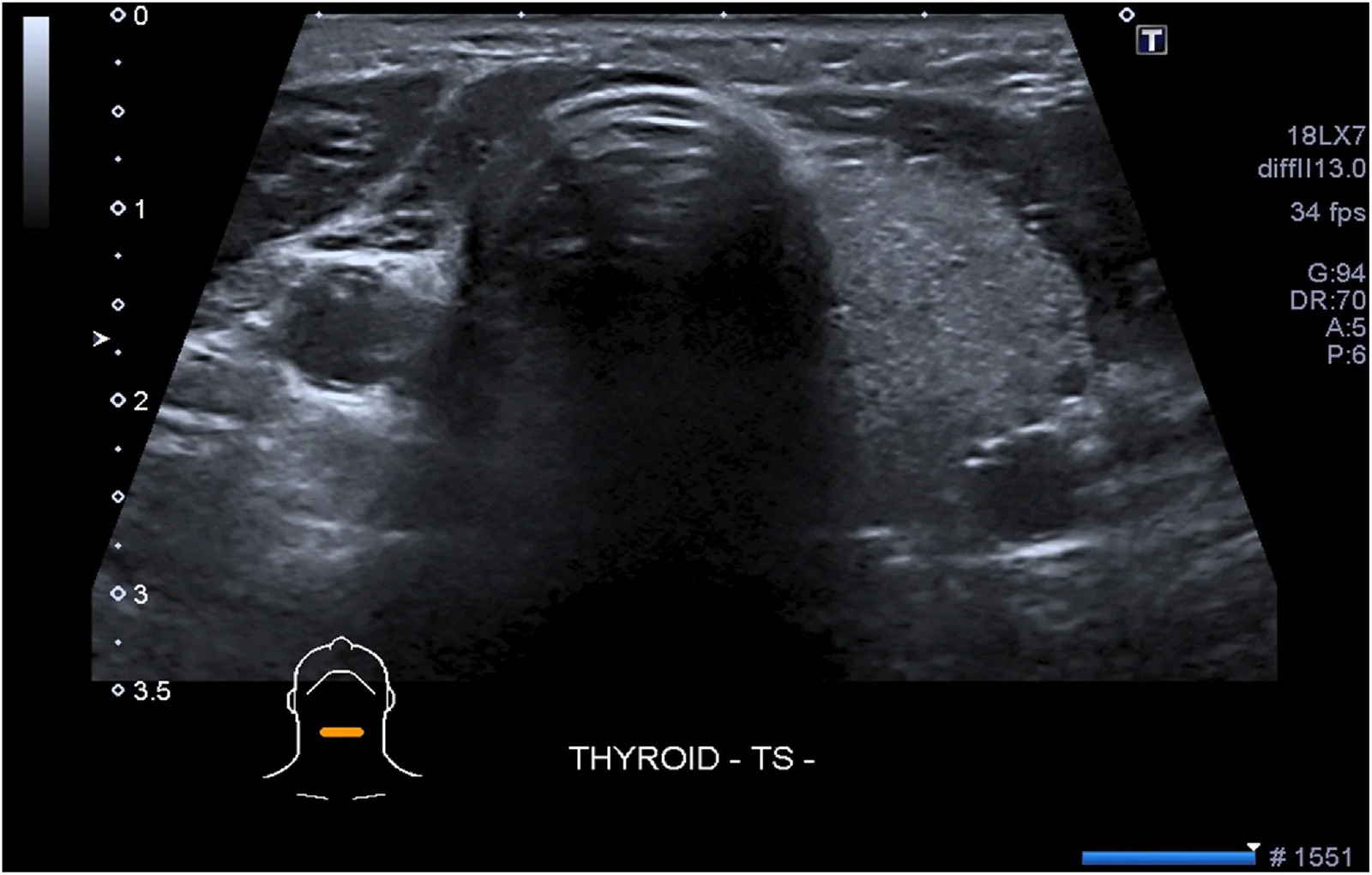
Thyroid ultrasound identifies a normal left lobe of thyroid with no focal lesion or suspicious malignant features. Previous right hemithyroidectomy with normal surgical bed.

Ultrasound of the neck also demonstrated a previous right hemithyroidectomy with no thyroid or abnormal tissue in the right thyroid bed. The left lobe of thyroid was normal with no sonographic features of Grave’s disease (Fig. 5).

As the patient had functioning thyroid tissue in the neck, the left breast lump was surgically excised as per the pathology request for more confirmatory tissue and after discussion with the multidisciplinary team. The final histology again identified ectopic benign thyroid tissue. No additional breast or mammogram surveillance was recommended (patient remained part of the national breast screening program). Post operatively the patient remained well with normal thyroid function and no breast concerns.

## Discussion

Ectopic thyroid tissue to the breast is very rare with only case reports published [[1], [2], [3],^6^,^10^,^11^]. The breast mass in this case was indeterminate, low suspicion of malignancy (2%-10% risk) BIRADS 4a. It is important to perform a breast biopsy as up to 30% of isoechoic breast masses can be malignant including lobular breast carcinoma, high grade breast tumors and metastases [12]. Primary thyroid cancer with metastasis to the breast is extremely rare with a few case reports identifying papillary, follicular and sarcoma metastatic phenotypes in the literature [[13], [14], [15]].

As ectopic thyroid tissue to the breast is so rare this was not considered initially in our case, In hindsight thyroid tissue can have this echogenic texture on sonographic imaging, although it is not necessarily possible to distinguish if benign or malignant [[13], [14], [15]] hence biopsy is paramount.

Once ectopic thyroid tissue is identified, functional imaging with technetium-99 m pertechnetate, iodine 123 or diagnostic dose of iodine-131 or can be used. The thyroid tissues takes up the radioisotope, identifying all sites of thyroid tissue in the body aiding management [16].

Our center used I-123 SPECT/CT for fused anatomical and functional imaging to precisely locate the functioning left thyroid tissue and the unifocal left breast ectopic tissue. Performing a thyroid ultrasound confirmed no malignant or background changes to the remaining thyroid or right thyroid bed.

If the ectopic breast thyroid tissue was the only functioning tissue, removal would have rendered the patient hypothyroid.

If there is malignant transformation of the ectopic tissue then imaging with Fluorine-18 fluorodeoxyglucose Positron Emission Tomography/Computed Tomography (F-18 FDG PET/CT) plays an important role in tumor evaluation, identifying distant disease, treatment response and tumor aggressiveness quantification. This can be used with either I-123 SPECT CT or I-131 to map and or treat the disease [17].

There is no consensus or formal guidance on the diagnosis and treatment of ectopic thyroid tissue in the breast. Usually, euthyroid patients will have ectopic tissue identified incidentally as in this case and management can be conservative for asymptomatic patients and benign histology [3]. Hypothyroid patients with ectopic thyroid tissue should be receive thyroid hormone replacement therapy, and this most commonly occurs in childhood [18].

Hyperthyroidism from ectopic thyroid tissue is less common than hypothyroidism. Ectopic thyroid tissue with features of Graves' disease has been reported in locations such as the tongue base, lateral neck, mediastinum, and small bowel mesentery including some with previous partial or total thyroidectomies [19].

Basaria et al. [19] reported thyrotoxicosis due to an ectopic mediastinal thyroid lesion and probably due to stimulation of the thyroid remnant thyroid-stimulating immunoglobulins (TSI) [19]. Our patient has a history of Graves' disease which although treated may have resulted in growth of a small focus of ectopic thyroid tissue to form a nodule in the breast. Surgical removal of the ectopic thyroid lesion in the breast was preferred in case the ectopic breast tissue was stimulated in the future by TSI. As she had a normal, functioning left thyroid remaining, she was unlikely to become hypothyroid.

A few studies have proposed embryologic links between the breast and ectopic thyroid tissue with women having an increase in co-occurrence cancer of these 2 organs [20]. Although clinicians should be aware of this association of thyroid and breast cancer the cause of the increase in risk remains unclear. Theories include common hormonal receptor pathway involving estrogen and TSH and shared genetic susceptibility [21]. These findings highlight the need for further investigation into the molecular pathways that may contribute to the occurrence of ectopic thyroid tissue in the breast.

## Conclusion

This report describes a rare case of benign ectopic thyroid tissue in the breast after a history of previous Graves’ disease and benign left thyroidectomy. Many patients (as in this case) do not describe thyroid disease signs and symptoms. It is paramount to exclude malignancy from primary breast disease, from thyroid malignancies or other metastatic disease with a breast core biopsy and thyroid immunohistochemistry. Functional and anatomical imaging is needed to identify all sites of thyroid tissue including ectopic sites. Multidisciplinary working with endocrinology is needed to evaluate thyroid function and aid ongoing management.

## Patient consent

The authors confirm that written, informed consent for publication of this case is obtained from the patient.
